# Kinetic Energy of Tornadoes in the United States

**DOI:** 10.1371/journal.pone.0131090

**Published:** 2015-07-01

**Authors:** Tyler Fricker, James B. Elsner

**Affiliations:** Department of Geography, Florida State University, Tallahassee, Florida, USA; University of Vigo, SPAIN

## Abstract

Tornadoes can cause catastrophic destruction. Here total kinetic energy (TKE) as a metric of destruction is computed from the fraction of the tornado path experiencing various damage levels and a characteristic wind speed for each level. The fraction of the path is obtained from a model developed for the Nuclear Regulatory Commission that combines theory with empirical data. TKE is validated as a useful metric by comparing it to other indexes and loss indicators. Half of all tornadoes have TKE exceeding 62.1 GJ and a quarter have TKE exceeding 383.2 GJ. One percent of the tornadoes have TKE exceeding 31.9 TJ. April has more energy than May with fewer tornadoes; March has more energy than June with half as many tornadoes. September has the least energy but November and December have the fewest tornadoes. Alabama ranks number one in terms of tornado energy with 2.48 PJ over the period 2007–2013. TKE can be used to help better understand the changing nature of tornado activity.

## Introduction

On average about 1,000 tornadoes occur each year in the United States; more than in any other country [1]. A violent tornado can destroy an entire town in minutes making it vital to understand what factors contribute to this power. For example, according to the National Weather Service (NWS) the Joplin, Missouri (2011) tornado caused 161 fatalities and more than 1,000 injuries, while producing about $3 billion of insured losses in less than 20 minutes [2]. Similarly, the Tuscalossa, Alabama (2011) tornado caused 65 fatalities and over 1,000 injuries, while producing about $2 billion of insured losses in just over an hour [3].

Current understanding of tornado destruction comes largely from available data records. The Storm Prediction Center’s (SPC) severe weather database is the most extensive set of historical tornado records in the world. Information in the database is compiled from the National Weather Service’s (NWS) *Storm Data*, which includes all known tornado reports dating back to 1950. Each report in the database gives the tornado touchdown location (latitude and longitude), date, length and width of the damage path, and maximum damage rating on a scale from 0 to 5.

The frequency of tornadoes by damage rating is a way to compare tornado occurrences collectively. For instance, Elsner et al. [4] show that the number of tornadoes by damage category on days with at least one violent tornado (EF4 or EF5) follows an exponential rule. On average the number of tornadoes at the next lowest damage level is twice the number at the current level. But the damage rating is only one component of destruction. On average longer and wider tornadoes have higher damage ratings [5, 6] but there is large variability in path dimensions from one tornado to the next.

By multiplying the path area by the damage rating and summing over all tornadoes in a given day, Thompson and Vescio [7] define an index useful for comparing destruction on days with many tornadoes (outbreaks). The destructive potential index (DPI) accounts for the difference in energy between a short-track weak tornado and long-track violent tornado better than does the damage rating alone. Likewise, the cumulative tornado destruction index (TDI) is defined as the square of the product of a characteristic wind speed and the path width [8].

The DPI and TDI are useful for summarizing a collection of tornadoes (tornado days for the DPI and tornado years for the TDI) and for comparing outbreaks. Here interest centers on kinetic energy of individual tornadoes. An estimate of KE is made in Agee and Childs [8] using the median wind speed for each damage category. Since the category is based on the worst damage within the tornado path, the estimate can be considered the instantaneous highest kinetic energy. Moreover since only velocity is used in the formula the result is a mass-specific energy.

In this study an estimate of total kinetic energy (TKE) is made for all tornadoes in the SPC database from 2007 when the Enhanced Fujita (EF) scale became operational, through 2013. The method uses the fraction of the tornado path experiencing EF damage and a characteristic wind speed for each EF rating [9]. Since the fraction of the path at each EF rating is not available for tornadoes in the SPC database a model is used instead. The model is taken from a study prepared for the U.S. Nuclear Regulatory Commission (NRC) to assess the risk of a tornado at any location [10]. The motivation for using the NRC model arises from the fact that it results in estimates of TKE that match well with estimates of TKE derived from using observed fractions from a set of recent tornadoes [9]. The formula for per-tornado TKE uses a characteristic vortex height and air density so that the values are expressed in energy units of Joules (J) rather than J/kg.

## Materials and Methods

### Data

The SPC maintains the most readily available tornado database in the world. Tornado reports in the database extend back to 1950. Relevant to this study per-tornado information includes occurrence time, location, damage rating, path width, path length, injuries, fatalities, and property loss. Here all tornadoes between 2007 and 2013 are used. The start year of 2007 coincides with the adoption of the EF Scale by the NWS.

Post storm surveys of the destruction in the wake of a tornado allow engineers to rate the damage on a scale from zero to five. Historically the damage scale was related physically to the tornado wind speed [11, 12]. Today wind speed is related to the observed damage [13]. The estimated wind speed is a three-second gust at the location of damage based on indicators to structures and vegetation. The indicators include the degree of damage taking into account differences in construction quality [14]. For instance EF1 (category one on the Enhanced Fujita scale) damage corresponds to wind speeds between 38 and 49 m s^−1^ and EF4 damage corresponds to wind speeds between 75 and 89 m s^−1^ (derived EF scale).

A tornado’s EF rating is assigned based on the greatest damage observed within the tornado’s path [15]. The EF scale is consistent with the original F scale but it includes additional damage indicators and it expands on the degree of damage. The scale was adopted by the National Weather Service (NWS) in 2007. Studies have addressed the need for more reliable measures of tornado winds and the potential discrepancies between wind speeds estimated by radar and damage ratings [16].

Estimates of tornado energy are not directly available in the database. The goal of this paper is to improve the understanding of tornado climatology through two objectives. The first is to use the available information to compute total kinetic energy of each tornado in the database. The second is to use the estimated kinetic energy as a basis for an alternative tornado climatology involving energy rather than frequency.

### Method

The main idea of this paper is to distinguish individual tornadoes based on a metric of destruction that has physical units of energy. Estimates of tornado energy have been made previously following the formula in Schielicke and Névir [17]. In particular, Fricker et al. [9] estimate TKE for 18 tornadoes over the period 2011–2013 using damage path information available in the NWS’s Damage Assessment Tool (DAT). They do this by computing a weighted average of the squared midpoint wind speed from the corresponding EF rating where the weights are the fraction of total damage area by each EF rating (maximum EF rating and lower). The equation is

$$\begin{array}{c} \text{TKE}=\frac{1}{2}m\;\sum_{j=0}^{J}\;w_{j}v_{j}^{2}, \end{array}$$

where *J* is the highest EF rating, *v*_*j*_ is the midpoint wind speed for each rating (e.g., *v*_0_ = 33.8 m s^−1^, *v*_1_ = 44.0 m s^−1^, etc), *w*_*j*_ is the corresponding fraction of path area, and *m* is the tornado mass, which is estimated as air density (1 kg m^−3^) times the volume (total path area times height). The tornado’s height and the density of air are held constant, thus *m* is only a function of total path area.

Since there is no upper bound on the EF5 wind speeds, the midpoint wind speed is set at 97 m s^−1^ (7.5 m s^−1^ above the threshold wind speed consistent with the EF4 midpoint speed relative to its threshold). It is acknowledged that tornado height varies considerably perhaps by as much as a factor of 10 or more, but without other information it is fixed at 1 km.

Since tornado-specific fractions of path area by EF rating are not available in the SPC database, here the NRC model of the fractions is used instead (see Table 1). The percent area by EF rating is listed down the six right columns. For example a tornado with a maximum EF rating of two has EF0 damage over 61.6% of its path area, EF1 damage over 26.8% of its path area, and EF2 damage over 11.5% of its path area.

**Table 1:** NRC model of percent damage area by EF rating. Redrafted from Table 3-1 in Ramsdell and Rishel [10].

	Wind Speed	Maximum EF rating					
	[m s^−1^]	EF0	EF1	EF2	EF3	EF4	EF5
EF0	29–38	1	0.772	0.616	0.529	0.543	0.538
EF1	38–49		0.228	0.268	0.271	0.238	0.223
EF2	49–60			0.115	0.133	0.131	0.119
EF3	60–74				0.067	0.056	0.070
EF4	74–89					0.032	0.033
EF5	89						0.017

The NRC model combines a Rankine vortex with empirical estimates to determine the fraction of path area associated with each EF rating [10]. Fricker et al. [9] used these path fractions to compute TKE for the tornadoes in the DAT. They also computed TKE using the observed path fractions given by the EF rating contours in the DAT and found them to be highly correlated.

**Fig 1:**
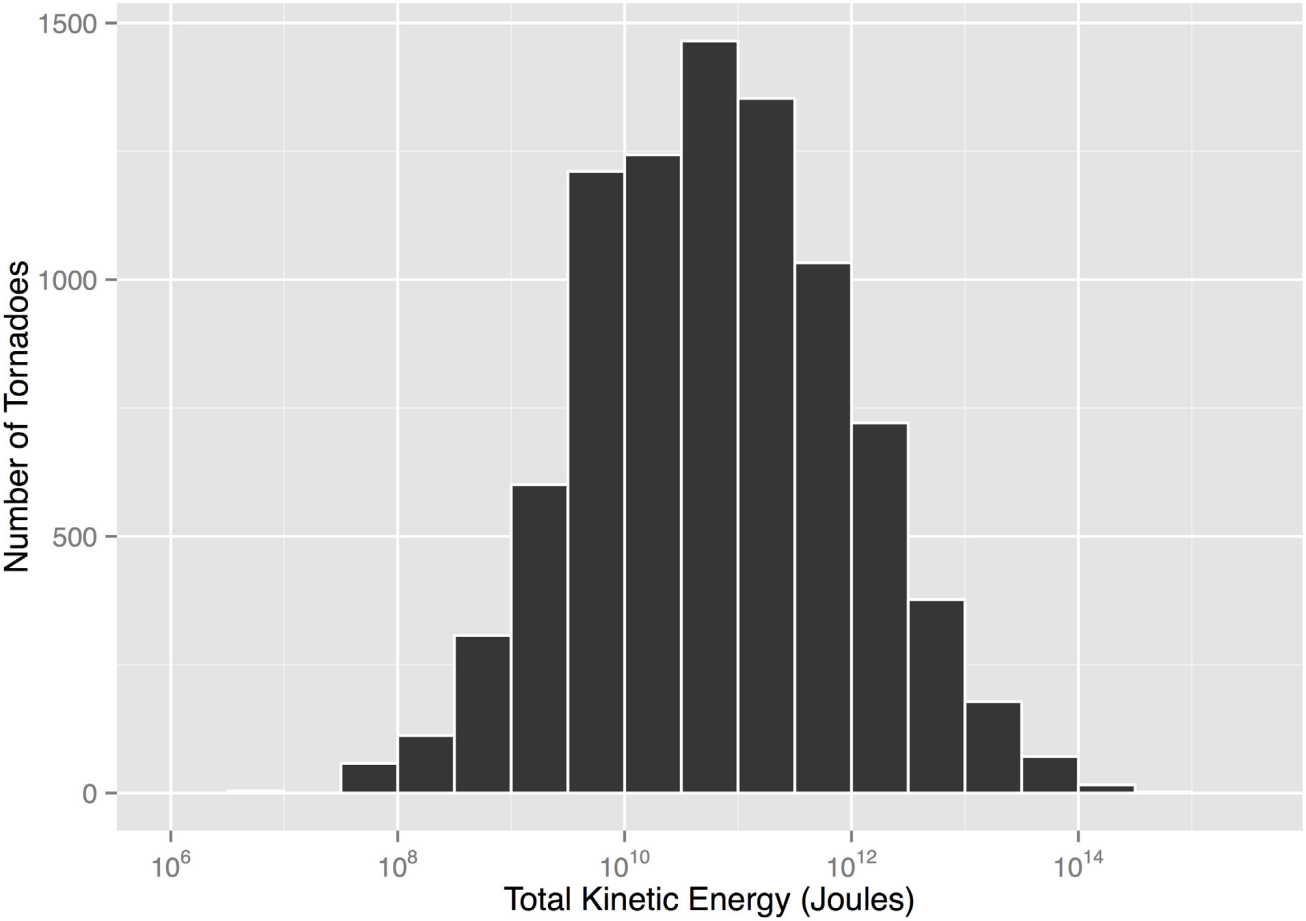
Distribution of total kinetic energy (TKE). TKE is computed for each tornado in the SPC database over the period 2007–2013.

TKE is computed for all 8752 tornadoes in the SPC database over the period 2007–2013. There are many more weak tornadoes (EF0) than strong ones (EF2 and higher) so the values are highly skewed with a median TKE of 62.1 gigajoules (GJ) and a interquartile range between 9.1 and 383.2 GJ. On a logarithmic scale the distribution is symmetric (Fig 1). Ten percent of the tornadoes over the period have TKE exceeding 1.97 terajoules (TJ or 10^12^ joules), five percent have TKE exceeding 5.53 TJ and one percent have TKE exceeding 31.9 TJ. The tornado with the most energy is the Tallulah-Yazoo City-Durant tornado of April 24, 2010 with a TKE of 516.7 TJ. It tracked over 240 km from Louisiana through Mississippi with a width of 2.82 km at its widest. It resulted in ten fatalities and 146 injuries. The most severe damage (EF4) occurred in the Mississippi counties of Yazoo and Holmes.

### Validation

The method to estimate TKE is validated by comparing resulting values with other indexes of tornado destruction and by correlating TKE with casualties and loss variables. This is done on a per-tornado basis. Per-tornado DPI is computed following Thompson and Vescio [7] as

$$\begin{array}{c} \text{DPI}=A\cdot (J+1), \end{array}$$

where *J* is the highest EF rating and *A* is the path area estimated by multiplying the path length by the path width. Path width is provided in the SPC database as the maximum width across the tornado path. Per-tornado TDI is computed following Agee and Childs [8] as

$$\begin{array}{c} \text{TDI}={\left(v_{J}\cdot W\right)}^{2}, \end{array}$$

where *v*_*J*_ is the midpoint wind speed for the highest EF rating (*J*) and *W* is the path width. Note that TDI and DPI are metrics that have never previously been computed on a per tornado basis.

Across all tornadoes in the database over the period 2007–2013, TKE correlates at a level of .99 with DPI indicating a very tight correspondence. In contrast TKE correlates at a level of .73 with TDI indicating a significantly lower correspondence. The correlation drops to .65 for the 66 violent tornadoes (EF4 and EF5).

A rank of the top ten tornadoes with the most energy shows the tight relationship between TKE and DPI (Table 2). The top six tornadoes by TKE match the top six by DPI. Only the long-tracked EF3 tornado of March 2, 2012 ranked tenth by TKE does not appear in the top ten by DPI. In stark contrast only three of the top ten ranked by TDI make it in the top ten by TKE. Missing from the definition of TDI is path length, which appears in the definition of both TKE and DPI. It is interesting to note that only one EF5 tornado makes it in the top ten list with seven of the top ten being rated as EF4. Five of the top ten tornadoes with the most energy occurred on April 27, 2011.

**Table 2:** Top ten tornadoes by TKE. The rankings are over the period 2007–2013. DPI is divided by square km and TDI is expressed in units of mass-specific energy times area.

	Max	TKE	DPI	TDI	DPI	TDI
Name/Location/Date	EF	[TJ]	[km^−2^]	[GJ m^2^ kg^−1^]	rank	rank
Tallulah-Yazoo City-Durant, LA (2010-04-24)	4	516.7	2651.6	53.1	1	4
Hackleburg-Phil Campbell, AL (2011-04-27)	5	353.7	2013.8	38.0	2	6
Tuscaloosa-Birmingham, AL (2011-04-27)	4	236.2	1212.2	37.8	3	8
Cordova, AL (2011-04-27)	4	202.7	1039.9	11.1	4	45
Argo-Shoal Creek-Ohatchee-Forney, AL (2011-04-27)	4	192.9	989.9	17.3	5	26
Clinton, AR (2008-02-05)	4	181.1	929.4	9.75	6	55
Vilonia, AR (2011-04-25)	2	179.4	684.8	21.4	10	20
Picher, OK (2008-05-10)	4	149.7	768.0	17.3	8	26
Smith-Jasper-Clarkem, MS (2011-04-27)	4	144.3	740.6	6.17	9	102
West Liberty, KY (2012-03-02)	3	142.7	620.8	9.5	11	57

The top five days with the most energy match the top five days with the highest DPI while the next five days of high energy match the next five high DPI days but in slightly different order. Seven of the top TDI days are in the top ten days with the most energy but not in the same order with the exception of April 27, 2011, which has the largest TDI, DPI, and TKE values.

Another validation of TKE as a useful measure of destruction is available by correlating it with fatalities, injuries, and losses (Table 3). TKE is positively correlated with the number of fatalities, the number of injuries, and the amount of loss. The highest correlation of .415 ([.401, .430] 90% confidence interval, CI) occurs with fatalities. DPI is also positively correlated with both casualties and losses and with slightly higher correlations although the uncertainty intervals overlap.

**Table 3:** Correlations between indexes of destruction and casualties and losses. The indexes include the total kinetic energy (TKE), the destructive potential index (DPI), and the tornado destruction index (TDI). The 90% confidence intervals are shown in parentheses.

Metric	Number of Fatalities	Number of Injuries	Loss Amount
TKE	.415 (.401, .430)	.394 (.379, .409)	.366 (.351, .381)
DPI	.445 (.430, .459)	.408 (.394, .423)	.390 (.375, .405)
TDI	.350 (.334, .365)	.332 (.316, .347)	.338 (.323, .354)

## Results

**Fig 2:**
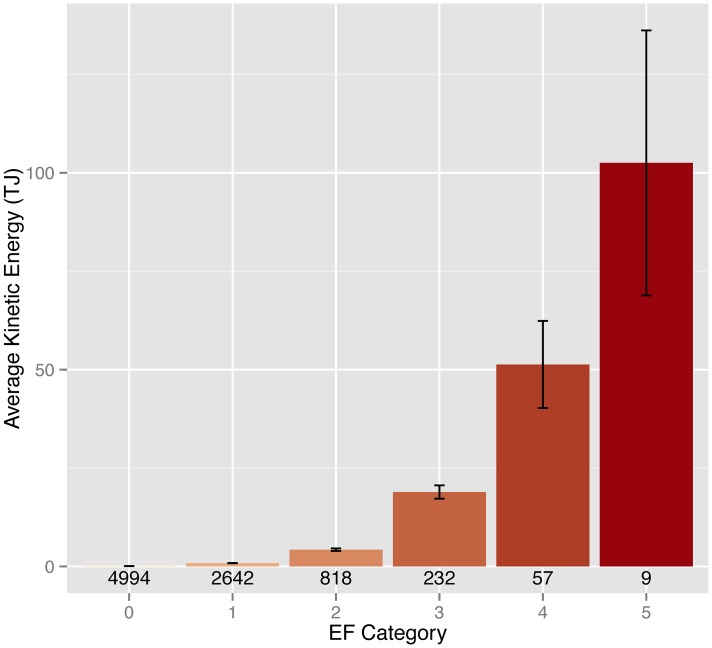
Average tornado energy by EF rating category.

In the previous section TKE as a metric of tornado destruction is defined with units of energy. Then it is shown that TKE has the same or greater explanatory power (statistically) as earlier indexes of destruction including DPI and TDI. Here variations in TKE are examined. Since energy is extensive, the per-tornado TKE values can be summed and averaged. For instance over the period 2007–2013 the average energy of the nine EF5 tornadoes is just over 100 TJ (Fig 2). The average energy of the 57 EF4 tornadoes is half of that at just over 50 TJ. The average energy of the 232 EF3 tornadoes is less than half of the EF4 and the average energy of the 818 EF2 tornadoes is significantly less than half of the EF3.

**Fig 3:**
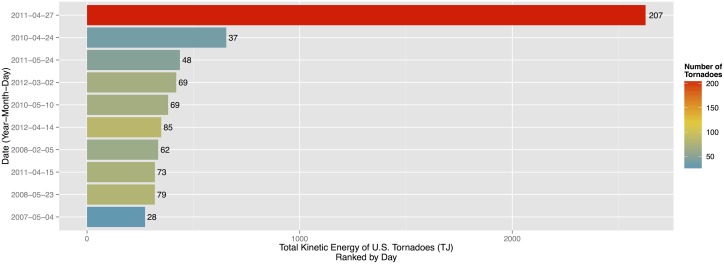
Top ten days ranked by daily TKE. The number of tornadoes is used as a color fill on the bars. Tornado counts are listed to the right of each bar.

Accumulating per-tornado TKE over all tornadoes in a day produces an estimate of daily kinetic energy. There are 1185 days with at least one tornado during the period 2007–2013. By a wide margin the day with the most energy is April 27, 2011 with more than 2.6 petajoules (PJ or 10^15^ joules) or four times the energy of the next most energetic day (Fig 3). The energy is accumulated over 207 tornadoes. The day with the next most energy is April 24, 2010 with 655 TJ from 37 tornadoes. Generally days with more tornadoes have more energy with a correlation of .707 ([.682, .730] 90% CI). Dividing the total daily energy by the number of tornadoes results in April 24, 2010 being the most efficient day at producing high-energy tornadoes with a per-tornado average TKE of 17.7 TJ followed by April 20, 2007 with a per-tornado average TKE of 15.0 TJ.

**Fig 4:**
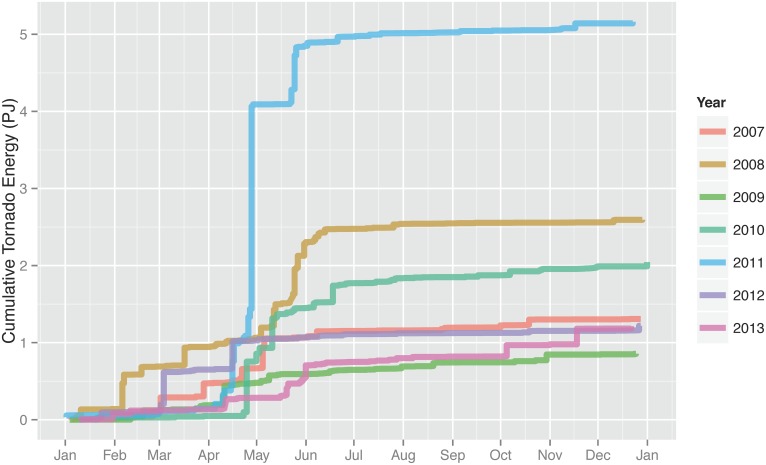
Daily aggregated tornado energy by year.

**Fig 5:**
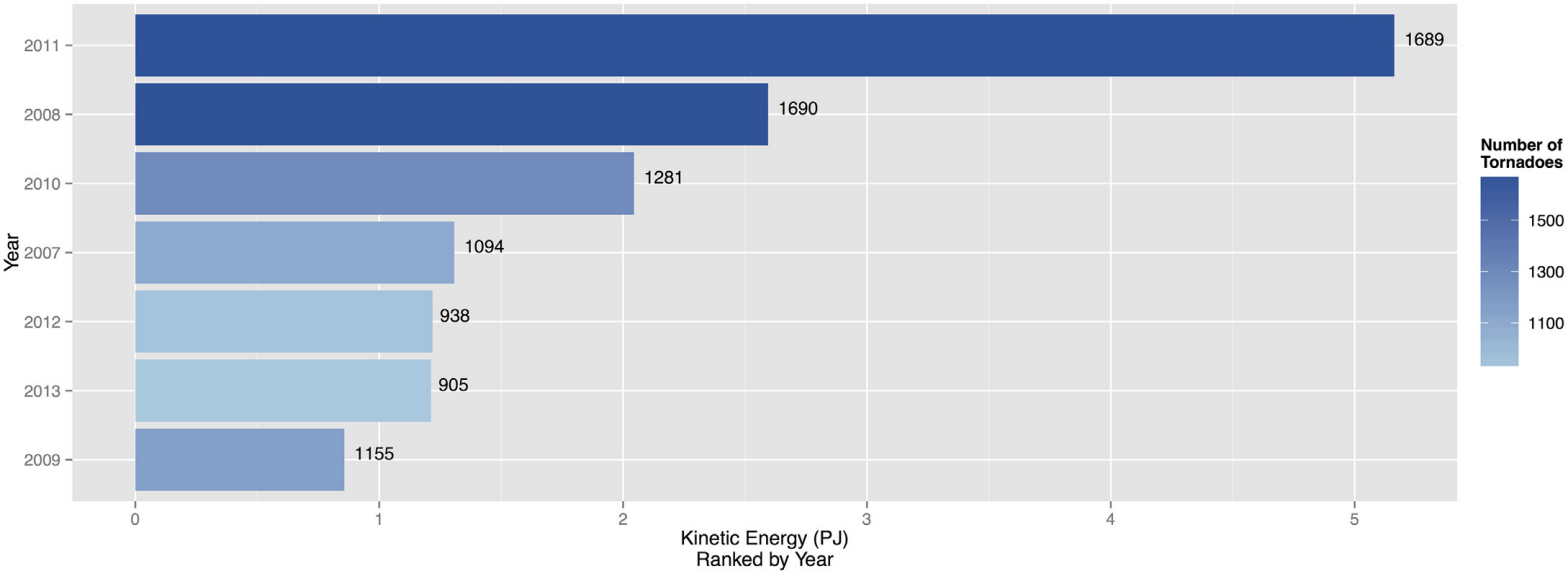
Annually aggregated tornado energy. Tornado counts are listed to the right of each bar.

The daily cumulative tornado energy changes by year (Fig 4). In general days in April and May tend to have the most energy but February was also quite active during 2008. The 2010 season started slowly but become very active from late April through mid June. The year with the most energy is 2011 and the year with the least is 2009 (Fig 5). Years with the most energy tend to have the most tornadoes although 2009 was an exception with relative high amount of energy from relative few tornadoes.

**Fig 6:**
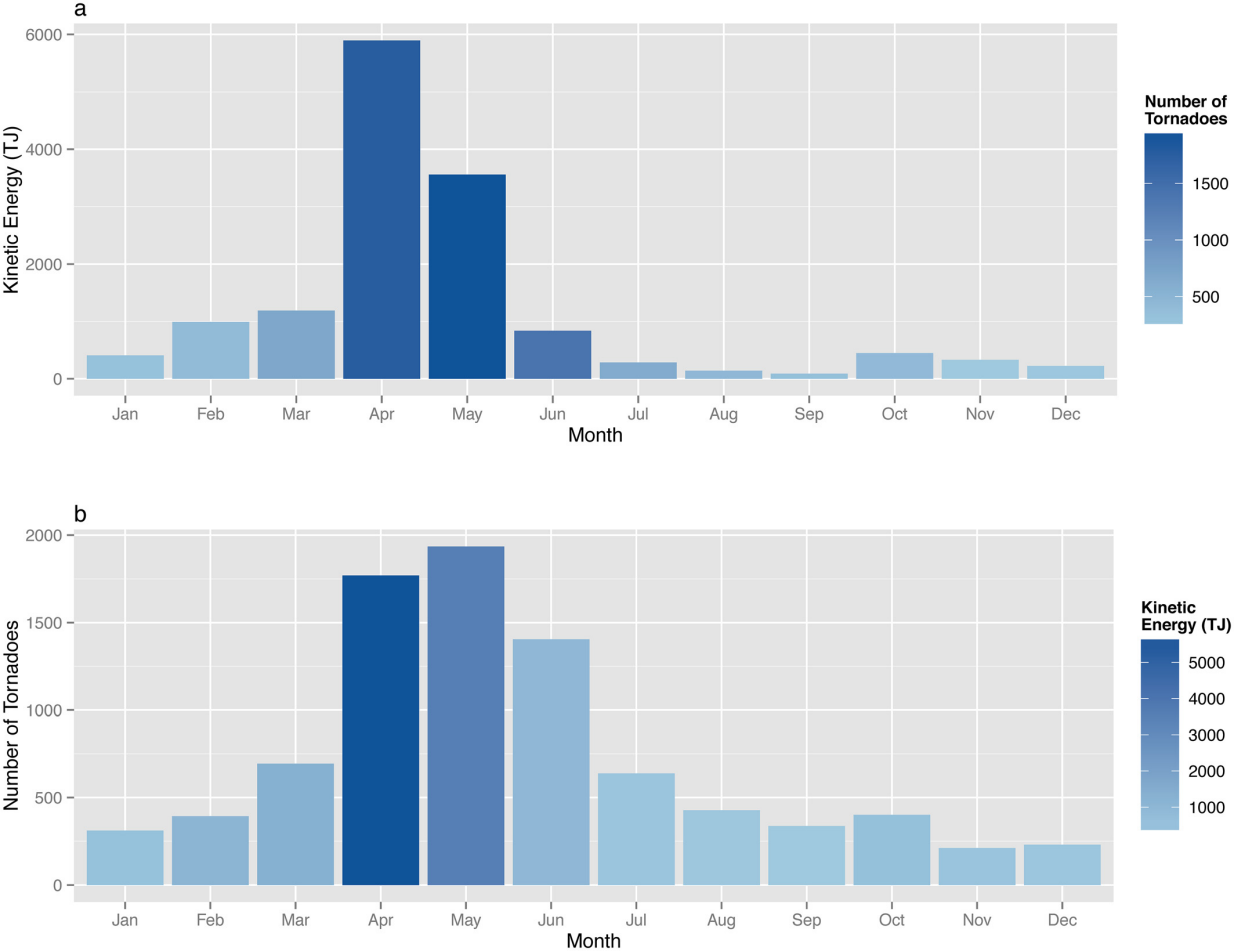
Tornado energy and frequency by month.

The seasonality of energy and frequency are similar with peaks from mid to late spring (Fig 6). However energy is relatively more concentrated during the year than is frequency. April has more energy than May with fewer tornadoes; March has more energy than June with half as many tornadoes. September is the month with the least energy but November and December are months with the fewest tornadoes.

**Fig 7:**
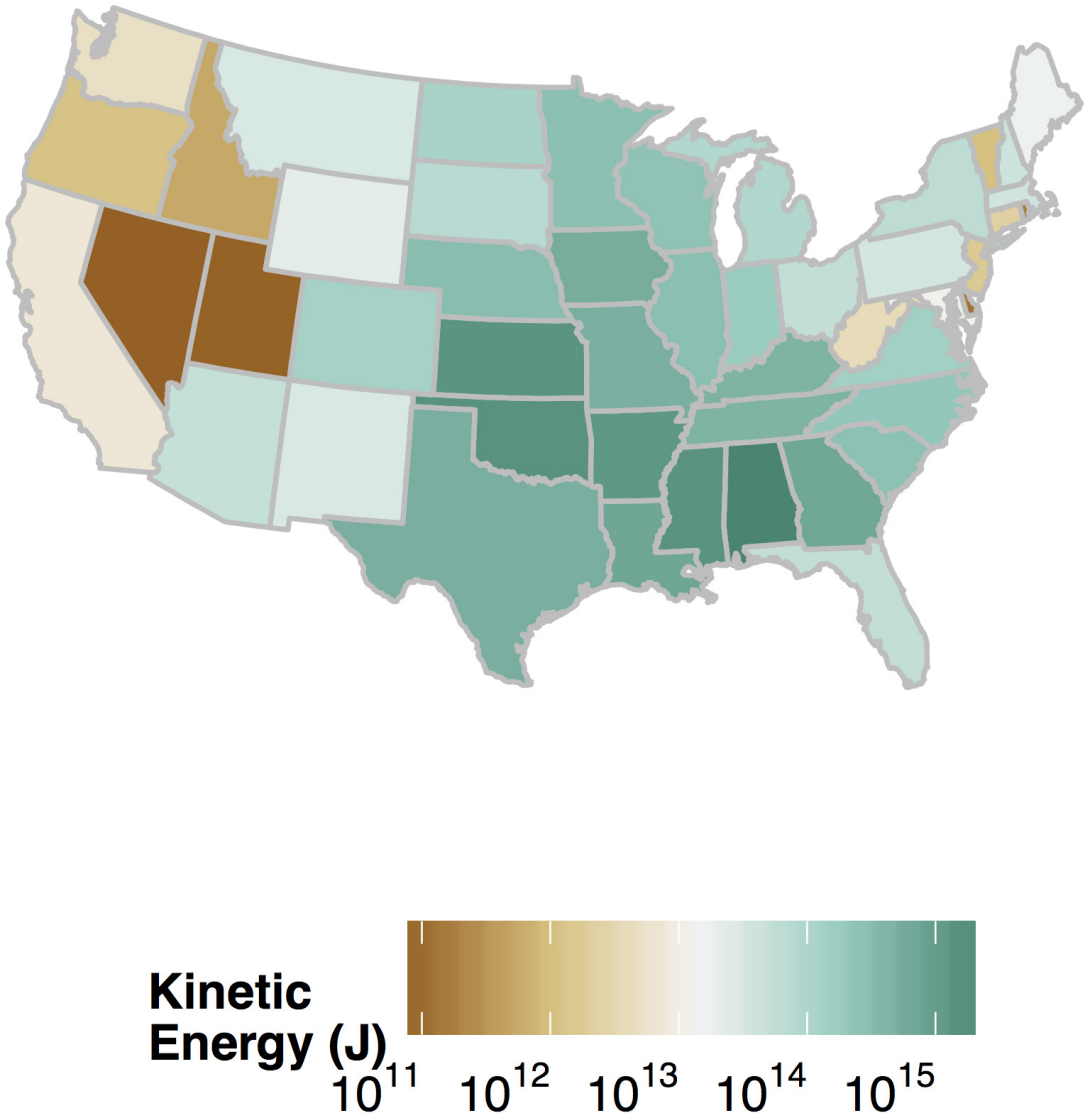
Tornado energy by state.

Tornado energy also varies regionally with the contiguous states of Kansas, Oklahoma, Arkansas, Mississippi and Alabama forming a large swath of high energy across the southern Great Plains southeastward into the deep South (Fig 7). The state having the most energy over the period is Alabama with a total of 2.48 PJ followed by Oklahoma with 1.45 PJ. The next four states in order of decreasing TKE are Mississippi, Kansas, Arkansas, and Louisiana. On a per-tornado basis, for those states with more than 100 tornadoes, the top six states in order are Alabama, Arkansas, Mississippi, Georgia, Oklahoma, and Louisiana. Five of the six are in the deep South.

## Discussion

The amount of destruction varies widely from one tornado to the next. To better understand this variability this study estimates the per-tornado total kinetic energy (TKE) for the 8752 tornadoes in the SPC database over the period 2007–2013. The main idea is to distinguish individual tornadoes based on a variable of destruction that has physical units of energy. The method uses the fraction of the tornado path experiencing EF damage and the midpoint wind speed for each EF rating. The fraction of the path is obtained from a model developed for the NRC that combines theoretical considerations with empirical data. An earlier study showed estimates of TKE from the model matched well those computed from recent tornado observations.

The TKE is validated as a useful metric of destruction by comparing it to other destructive indexes in the literature including the DPI and TDI. Additional validation is demonstrated by correlating it to casualties and loss estimates. TKE is highly correlated with DPI since they are both sensitive to path length and width in the same way (path area). As such TKE offers no statistical advantage over DPI as a single indicator of destruction for individual tornadoes. However, since TKE is based on physical theory and has units of energy it can be more easily understood by scientists outside of meteorology. Moreover, TKE has units of Joules (or more simply, J/kg) and can be directly compared with environmental energy variables like CAPE and helicity, which have the same units of J/kg. This is important toward the goal of improving understanding of the relationship between tornadoes and their parent-storm environment. Moreover, TKE can be spatially dis-aggregated. For example, if interest lies in the portion of energy within a county from a tornado passing through several counties, then the county-level TKE can be computed based on the contours by EF rating. In this way maps of TKE by area can be drawn accumulated by month, year, and decades.

The per-tornado TKE values are summed and averaged to produce an alternative climatology of tornadoes based on energy rather than frequency. Half of all tornadoes have TKE that exceed 62.1 GJ and a quarter have TKE that exceed 383.2 GJ. Five percent of the tornadoes have TKE that exceed 5.53 TJ. The tornado with the greatest amount of energy is the long-tracked EF4 Tallulah-Yazoo City-Durant tornado of April 24, 2010 with a TKE of 516.7 TJ resulting in ten fatalities and 146 injuries. The single day with the most energy is April 27, 2011 with more than 2.6 PJ from 206 tornadoes.

The average energy of the nine EF5 tornadoes is just over 100 TJ and the average energy of the 57 EF4 tornadoes is half of that at just over 50 TJ. The average energy of the EF3 tornadoes is less than half of the EF4 and the average energy of the EF2 tornadoes is much less than half of the EF3. Tornado energy peaks from mid to late spring similar to the peak in tornado frequency. Key new findings are that April has more energy than May with fewer tornadoes; March has more than June with half as many tornadoes. September is the month with the least energy but November and December are months with the fewest tornadoes.

Another key finding is that Alabama ranks number one in terms of tornado energy with a total of 2.48 PJ followed by Oklahoma with 1.45 PJ. The next four states in decreasing order are Mississippi, Kansas, Arkansas, and Louisiana. On a per-tornado basis, for those states with more than 100 tornadoes, five of the top six states are in the South. The spatial pattern of TKE at this scale suggests a single tornado threat region encompassing both the traditional alley of the Great Plains and the Dixie alley of the South [18]. The problem of frequent tornadoes in the Great Plains is a well understood and researched threat compared with the threat of strong tornadoes in the South.

Finally, recent research shows a future with more days when high wind shear coincides with high values of convective available potential energy [19] indicating the possibility for more powerful tornadoes. An index of destruction with units of energy on a continuous scale will help scientists to better understand the changing nature of tornado activity.
